# Subjective Assessments of Quality of Life Are Independently Associated with Depressive Symptoms among Older Adults Enrolled in Primary Care in Chile

**DOI:** 10.3390/jpm12071063

**Published:** 2022-06-29

**Authors:** Ximena Moreno, Hugo Sánchez, Martín Huerta, Ximena Cea, Carlos Márquez, Cecilia Albala

**Affiliations:** 1 Facultad de Psicología, Universidad San Sebastián, Santiago 7510157, Chile; 2 Institute of Nutrition and Food Technology, Universidad de Chile, Santiago 7810000, Chile; hugo.sanchez@hurtadohosp.cl (H.S.); cmarquez@inta.uchile.cl (C.M.); calbala@uchile.cl (C.A.); 3 Integrative Medicine Unit, Centro de Diagnóstico y Tratamiento, Hospital Sótero del Río, Servicio de Salud Metropolitano Sur Oriente, Santiago 8207257, Chile; martinhterapias@gmail.com; 4 Facultad de Enfermería, Universidad Andrés Bello, Santiago 8370251, Chile; ximenacea@gmail.com; 5 Facultad de Medicina, Universidad de Chile, Santiago 8380453, Chile

**Keywords:** depression, aging, primary care, older adults, quality of life, health status, mental health

## Abstract

In Chile, depressive symptoms are highly prevalent among Chilean older adults, and research that examines the factors associated with them is scarce. This study aimed to determine if subjective assessments of quality of life are associated with positive screen for depressive symptoms among older adults enrolled in primary care in Chile. The participants of the study were people aged 70 years or more enrolled in primary care centers in three Chilean cities. The 15-item Geriatric Depression Scale was used to determine depressive symptoms. Multivariate logistic models were used to determine the associations. Overall, 17.28% men, and 26.47% women (*p* = 0.003) screened positive for depression. Subjective assessments of quality of life, including self-perceived health, memory, quality of life, and pain, were associated with a positive screen for depression. Only 17.65% of men and 43.55% of women who screened positive for depressive symptoms reported a diagnosis of depression. Assessments of quality of life in health checks of older adults in primary care could contribute to narrow the diagnosis and treatment gap by improving the ability to identify those who are more likely to experience depressive symptoms.

## 1. Introduction

According to the World Health Organization (WHO), depression is the most common mental health problem, reaching the highest prevalence among older adults, affecting approximately 5.5% of men and 7.5% of women between 55 and 74 years of age [1]. Depressive disorders are the leading mental health cause of years of life lived with disability and disability-adjusted life-years among men and women aged 50 years and older [2]. Although effective treatments for depression exist, underdiagnosis of depression among older adults has been reported in different regions of the world [3,4]. Depression in old age is associated with health status, including chronic diseases, polypharmacy, and disability [5,6], and with an increased risk of mortality [7]. With respect to sociodemographic characteristics, depression disorders are more frequent among women of all ages, but the factors involved in this difference in old age are not well-understood [8]. Furthermore, several studies have found no association between gender and depression in older adults [5]. Associations with age, education, marital status, and area of residence have been suggested, but the results are heterogeneous [5,9,10].

A systematic review observed that depression is also associated with global and health related quality of life in older adults and that greater severity of depression increased the likelihood of poor quality of life [11]. The impact of depression on quality of life in old age is stable over time, as it has been reported in prospective studies in Europe, Asia, and Latin America [12,13,14] Quality of life refers to how the position in life is perceived by an individual in the context of a specific culture and value systems and in relation to subjective goals, expectations, standards, and concerns [15]. Global quality of life is a multidimensional concept, including feelings of well-being, life satisfaction, and the subjective evaluation of health, social, economic, and spiritual aspects of life [11]. 

The number of studies about late-life depression in Chile is limited, but the results show that the prevalence of depressive symptoms is high and that above 64% of older adults with mild to severe symptoms of depression have not received a diagnosis [16,17]. The adverse outcomes associated with depression in old age stress the need to determine which groups of older adults are more likely to experience depressive symptoms in Chile. According to population studies, the prevalence of major depressive disorder among Chilean older adults has been estimated between 3.6 and 5.0 percent considering the DSM-IV and ICD-10 criteria [18,19]. Positive screening for severe depression among older women in Chile ranges between 5.4 and 6.3 percent and between 7.4 and 12.9 percent among older men, as reported by previous studies [16,17,20]. As a response to the high burden of disease attributable to depression, the Chilean national program for detecting, diagnosing, and treating depression was launched by the beginning of the century, and it was implemented in primary care by 2004 [21]. In 2005, depression was included in the Regime of Explicit Health Guarantees, aimed at providing universal access, opportunity, financial protection, and quality of care for 80 prioritized health conditions [22]. In the case of older adults enrolled in primary care in Chile, they undergo, at least once a year, an exam to identify people at risk of dependence, which includes a screening for depressive symptoms [23]. However, as described previously, a high proportion of older adults in Chile who screen positive for depression are not diagnosed [16,17].

Research about the factors associated with depression in older adults in Chile is scarce. Associations with gender, age, education, living alone, region of residence, multimorbidity, disability, pain, feelings of loneliness, and spirituality have been found [16,17,24,25,26]. Only two studies considered variables from different domains, including sociodemographic factors and health status indicators [16,17]. No previous study has analyzed a set of subjective assessments of quality of life along with sociodemographic and health variables. Furthermore, considering that older adults mainly consult and receive routine treatment in primary care [27], it is important to describe the factors associated with depression among people enrolled in primary care to better characterize this group. 

The aim of this study was to determine if subjective assessments of quality of life were independently associated with a positive screen for depression among community-dwelling older adults enrolled in primary care in Chile. We also explored if quality of life assessments were associated with having received a diagnosis of depression.

## 2. Materials and Methods

### 2.1. Participants and Setting

**Figure 1:**
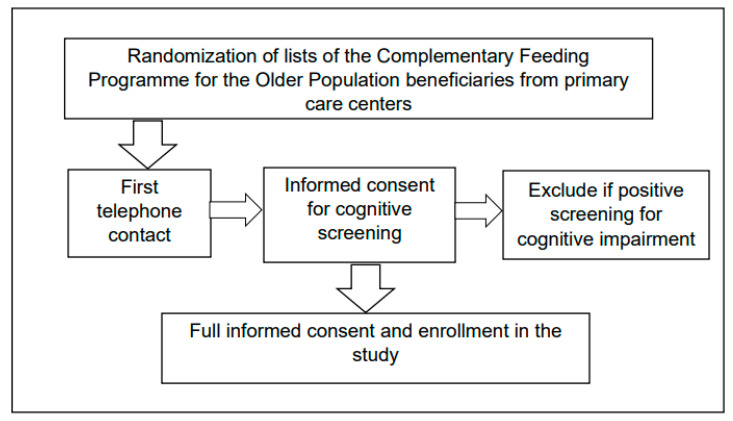
Diagram of the recruitment process.

The data analyzed were collected in a study aimed at assessing the optimal levels of vitamin D and B12 in a nutritional supplement distributed in primary care centers in Chile to improve their cognitive and physical functionality [28]. Methodological details have been previously described [29]. Briefly, the outcomes of the study were: deficit of vitamin B12 (defined as vitamin B12B12 < 221 pmol/mL; estimated prevalence of 41.0%), deficit of vitamin D (defined as vitamin D < 50 nmol/dL; estimated prevalence of 45.4%), walking ability (assessed with the timed-up-and-go test; estimated mean = 10.8 s; standard deviation = 5.5 s), and cognitive status (assessed with the Mini Mental State Examination; estimated mean = 27.6). The study detected a change of 20 percent points in deficit of vitamin B12, 30 percent points difference in deficit of vitamin D, 2 s difference in walking ability, and 1 point difference in cognitive status score; considering an α = 0.05, β = 0.2, and an estimated loss of 15%, the estimated sample size was 300. The inclusion criteria were age of 70 or more years, being enrolled in primary care, and being beneficiaries of the Complementary Feeding Programme for the Older Population (PACAM). The exclusion criterion was a positive screen for cognitive impairment assessed with the Mini Mental State Examination and the Pfeffer Functional Activities Questionnaire [30]. The recruitment process is described in Figure 1. Participants were randomly selected from primary care center registration lists of people aged 70 or more years from three cities located in different regions of Chile. One city is in the furthest north (Iquique), another in the furthest south (Punta Arenas), and the other is the capital city (Santiago) located in central Chile. These cities differ in population density, climate features, historical and cultural elements, average household income, poverty, and the number of medical specialists per 1000 inhabitants [29,31]. 

Face to face interviews were carried out by health professionals, who collected sociodemographic and health information. A total of 796 people participated in the study. Of them, 777 (97.61%) who had complete data on depression screening were included in our analyses. 

### 2.2. Measurements and Variables

The outcome of the study was depressive symptoms. This variable was measured with the 15-item Geriatric Depression Scale (GDS-15), which is a short form of the 30-item original depression screening test for older adults (Park and Kwak, 2021). It includes 15 closed-ended questions with respect to how the person has felt during the past week. The possible answers are “yes” or “no”. The total score of the test ranges from 0–15. The recommended cutoff score to detect major depressive disorder among older adults is ≥5, with a pooled sensitivity of 0.89 and a specificity of 0.77 [32]. 

Depression diagnosis was self-reported. The question used was: “Has a doctor ever told you that you have depression?”.

With respect to quality of life, questions about subjective assessment of different dimensions were used, including health, age, pain, quality of life, memory, and social support:

- To assess self-rated health, the question employed was: “In general, would you say your health is: Excellent, very good, good, fair, poor?”. For analytical purposes, the answers were collapsed into “good” (excellent, very good, and good) and “less than good” (fair and poor).
- Pain was assessed with the question: “Did you have pain in any part of your body, during the last four weeks)?”. A six-point scale was used to answer this question, ranging from 1 (no, no pain) to 6 (yes, very much).
- Self-perceived age was assessed with the question: “Some people of your age feel old, some feel middle aged, and some feel young. How do you feel?”. The possible answers were: young, middle-aged, old, and very old. The answers were collapsed into “not old” (young and middle-aged) and “old” (old and very old).
- A general question about quality of life was used: “How would you say is your quality of life in the present?”. This question included the following answers: excellent, very good, good, fair, poor. The answers to this question were collapsed into “good” (excellent, very good, and good) and “less than good” (fair and poor).
- Self-rated memory was assessed with the question: “How do you rate your memory in the present?”. The response categories were: excellent, very good, good, fair, and poor. Two collapsed categories were used in the analysis: “good” (excellent, very good, and good) and “less than good” (fair and poor).
- To assess self-perceived social support, the question employed was: “If you need material support, company, or advice, do you have someone you can turn to?”. The possible answers were “yes” or “no”.

Sociodemographic variables included in the analyses were: age (in years), gender, years of education (categorized as none, less than 8, 8–11, and 12 or more), marital status (married or with a partner, single, separated or divorced, widowed), and living alone. 

Health variables were:

- Self-reported number of chronic diseases, including hypertension, diabetes, Parkinson’s disease, coronary heart disease, chronic obstructive pulmonary disease, stroke, cancer, osteoarthritis, and cataracts.
- Disability was defined as the inability to perform one or more basic activities of daily living, including walking, bathing, getting dressed, eating, using the toilet, and getting in or out of bed.
- The question: “How many medications per day are you taking?” was used to assess the number of medications.

### 2.3. Analyses

To describe the sample, percentages are reported for categorical variables, and means for continuous variables. *p*-values were calculated to determine the difference in the distribution of each variable between men and women, using the chi-square test or the *t*-test to compare two means when corresponding.

A purposeful selection of variables to build the multivariate logistic models was used. The unadjusted association between each variable and positive screen for depression was calculated. The variables that were associated with the outcome were included in the adjusted logistic model. The same criterion was used to select the covariates to include in the multivariate logistic model with diagnosis of depression as the outcome. The level of significance was specified at 0.05. Fully adjusted logistic models for positive screening for depression and for depression diagnosis are reported, with 95% confidence intervals.

Stata 14 (StataCorp, College Station, TX, USA) was the software used to carry out all the analyses

### 2.4. Ethical Considerations

The protocol of the study was approved by the ethics committee of the Institute of Nutrition and Food Technology, Universidad de Chile. All participants signed an informed consent.

## 3. Results

### 3.1. Participants Description

The mean age of the sample was 77.16 years (SD = 5.34). The age range was 26 years (from 70 to 96 years). In total, 61.26% of the sample were women. The characteristics of the sample are shown in Table 1. There were no gender differences in age, years of education, and city distribution. Only 12.52% of the sample had completed their secondary education. Men were more likely to be married (78.74%) compared with women (46.53%). A higher proportion of women were unable to perform one or more basic activities of daily living (12.87% vs. 4.33% of men). On average, women took more medications (4.31 than men (3.83). More than half of women (53.26%) rated their health as less than good compared to 36.88% of men in the same situation. Women reported a higher average level of pain than men. Positive screen for depression was observed in 22.91% of the sample (17.28% of men, 26.47% of women). Self-reported diagnosis of depression was also less frequent in men (6.69%) than in women (24.52%), with a frequency of 17.85% in the whole sample. 

Only 36.0% of people with a positive screen for depression had received a diagnosis of depression according to their self-report (17.65% of men, 43.55% of women, *p* = 0.001).

### 3.2. Factors Associated with a Positive Screen for Depression

As observed in Table 2, people who lived in the northern city (OR = 0.40, 95% CI 0.21–0.76) were less likely to screen positive for depression compared to people living in the capital city. There was no statistically significant association with another sociodemographic variable. People who were unable to perform one or more basic activities of daily living had more than four times the likelihood of a positive screen for depression (OR = 4.45, 95% CI 2.31–8.54). Less than good self-rated health (OR= 2.23, 95% OR 1.37–3.63), a higher level of pain (1.19, 95% CI 1.02–1.38), less than good self-perceived memory (OR = 1.67; 95% CI 1.07–2.62), and a less than good self-perceived quality of life (OR = 2.44, 95% CI 1.58–3.76) were associated with a positive screen for depression.

### 3.3. Factors Associated with Self-Reported Depression Diagnosis

Table 3 shows the adjusted association between self-reported diagnosis of depression and the covariates included in this model. Women were more than three times more likely to be diagnosed with depression compared to men (OR = 3.30, 95% CI 1.85–5.87). Being widowed was associated with having received a diagnosis of depression (OR = 2.01, 95% CI 1.24–3.26). Disability (OR = 2.11, 95% CI 1.10–4.07), higher number of medications (OR = 1.23, 95% CI 1.10–1.38), and higher level of pain (OR = 1.27, 95% CI 1.08–1.50) increased the likelihood of depression diagnosis. 

## 4. Discussion

Subjective assessments of different dimensions of quality of life were associated with a positive screen for depression. This association was observed for self-rated health, level of pain during the last month, self-perceived age, self-perceived memory, and self-perceived quality of life. The variables associated with positive screen for depression were not the same as those associated with self-reported diagnosis of depression. People with less positive assessments of their quality of life and with disability were more likely to screen positive for depression independently of their gender and age. With respect to diagnosis of depression, being women and widowed increased the likelihood to report this diagnosis. Disability, number of medications, and level of pain during the last month were also associated with this outcome.

Previous research has reported an association between gender and a positive screening for depression in older adults [8]. In our study, this association did not persist when considering the association with variables related to health status and quality of life. However, gender was associated with self-reported diagnosis of depression even after controlling for the association with health status and perceived quality of life. There was also a discordance between positive screen for depression and diagnosis, with self-reported diagnosis of depression in less than half of women and less than one-fifth of men who screened positive for depression. Previous studies have reported a gap between depression and diagnosis in older adults, particularly in men [16,17,33,34]. A study carried out among a sample of older adults enrolled in primary care in Chile, who had been monitored in the context of the Annual Preventive Medical Evaluation of the Elderly, found that the coverage of health evaluation by a physician due to a positive screen for depression reached only 14.6% in 2016 [35]. Considering that the highest rate of suicide is found among older men [36,37], efforts should be made to further understand underdiagnosis and undertreatment among them.

Disability was associated with a positive screen for depression and self-reported diagnosis of depression. These variables had been previously identified as a factor associated with depression [5]. No other objective measure of health status, apart from disability, was associated with a positive screen for depression. However, subjective assessments of health status (self-rated health, level of pain during the last month, and self-perceived memory) were independently associated with this outcome. Self-reported diagnosis of depression was associated with the number of daily medications, as previously reported in other studies [5,6]. People enrolled in primary care who report taking more medications probably have more health checks and are more frequently monitored by the health care staff. This might increase the chances to screen or detect depressive symptoms among them. Primary care centers in Chile also have annual goals associated with monitoring the health status of people aged 65 or more years to determine their risk of dependency with the Annual Preventive Medical Evaluation of the Elderly [23]. However, the difficulties of primary care centers to meet the health needs of older adults in Chile have been documented [37], and the coverage of this annual evaluation reaches only 40% [38].

Some aspects that should be considered and further explored in future research are the possibility of a higher under-report of depression diagnosis among men and a reluctance towards mental-health-treatment seeking. There is a social stigma about mental health problems in general [39,40] in the primary care context [41] and in older adults with depression [42], which is a barrier for mental health treatment seeking among older adults [43]. Furthermore, some studies have reported that men have a more negative view about male depression [44], which could contribute to a lower detection of depression among them in primary care settings.

Our results suggest that the region of residence should be considered when analyzing factors associated with a positive screen for depression. People who lived in the northern city were less likely to screen positive for depression compared with people from Santiago even after considering quality of life variables. Although both cities correspond to urban areas, Santiago, the capital city, is very different from Iquique in terms of population density and poverty distribution. Regional differences in the epidemiology of mental health problems in older adults should be studied to determine the need of context specific interventions.

This study used 5 as the cutoff score to determine positive screen for depression, as suggested by a meta-analysis [32]. This is the most frequent cutoff score, but when interpreting our results, it is important to consider that some studies suggest that the cutoff score could vary depending on the cultural context [45]. A Brazilian study found that a cutoff of 6 had a higher sensitivity and specificity to detect major depressive disorder among older adults free of cognitive decline [46]. In Chile, only the GDS-5 has been validated [47]. The optimal cutoff score for the GDS-15 among the Chilean population should be determined.

This study has some limitations. The sample was not nationally representative, and cognitive decline was an exclusion criterion. However, primary care is the most frequent setting where older adults seek mental health care [27,43], and our results are a contribution to characterize the factors associated with depression in older adults enrolled in primary care. Positive screening for cognitive impairment was an exclusion criterion. Previous research has shown that depression is associated with cognitive impairment in old age [48]; hence, this association should be addressed by future studies. 

In our study, self-assessments of quality of life were independently associated with positive screen for depression in older adults. Assessments of quality of life in health checks of older adults in primary care could contribute to narrow the diagnosis and treatment gap by improving the ability to identify those people who are more likely to experience depressive symptoms.

## Figures and Tables

**Table 1:** Characteristics of the sample.

	Total (*n* = 796)	Men (*n* = 309)	Women (*n* = 487)	*p*-Value
Age %				0.750
70–74	38.61	39.87	37.82	
75–79	31.66	30.23	32.56	
80–84	19.18	20.27	18.49	
85+	10.55	9.63	11.13	
City %				0.127
North	23.55	26.91	21.43	
Center	39.64	35.88	42.02	
South	37.21	36.55	37.2	
Years of education %				0.182
0	5.55	4.01	6.51	
<8	57.42	54.85	54.85	
8–11	24.52	27.42	27.42	
12+	12.52	13.71	13.71	
Marital status %				<0.001
Married	59.02	78.74	46.53	
Single	7.09	2.66	9.89	
Divorced	5.03	3.65	5.89	
Widowed	28.87	14.95	37.68	
Number of chronic diseases %				0.137
0	5.92	6.98	5.25	
1–2	53.67	56.81	51.68	
3+	40.41	36.21	43.07	
Disability %				<0.001
Yes	9.56	4.33	12.87	
Number of medications				0.003
Mean (SD)	4.12 (2.18)	3.83 (2.21)	4.31 (2.15)	
Self-rated health %				<0.001
Good	53.09	63.12	46.74	
Less than good	46.91	36.88	53.26	
Pain (1–6 scale)				<0.001
Mean (SD)	3.42 (1.59)	3.01 (1.55)	3.69 (1.55)	
Self-perceived age %				0.585
Not old	81.03	82.00	80.42	
Old	18.97	18.00	18.97	
Self-perceived memory %				0.772
Good	47.16	46.51	47.58	
Less than good	52.84	53.49	52.42	
Quality of life %				0.347
Good	67.65	65.67	68.91	
Less than good	32.35	34.33	31.09	
Self-perceived social support %				0.503
Yes	87.98	87.00	88.61	
No	12.02	13.00	11.39	
Positive screen for depression %				0.003
Yes	22.91	17.28	26.47	
No	77.09	82.72	73.53	
Self-reported diagnosis of depression %				<0.001
Yes	17.62	6.69	24.52	
No	82.38	93.31	75.48	

**Table 2:** Adjusted odds ratio of positive screen for depression.

Variable (Reference)	OR	95% Confidence Interval
Gender (men)	1.06	0.37–1.67
Age (70–74 years)		
75–79	0.78	0.37–1.67
80–84	0.98	0.25–3.74
85+	0.97	0.11–8.26
Region (center)		
North **	0.40	0.21–0.77
South	0.68	0.41–1.13
Years of education (12+)		
0	1.10	0.37–3.28
1–7	0.88	0.42–1.84
8–11	0.88	0.40–1.94
Marital status (married)		
Single	1.12	0.46–2.72
Divorced	1.41	0.48–4.10
Widowed	1.47	0.87–2.49
Number of chronic diseases (0)		
1–2	2.44	0.53–11.20
3+	4.50	0.95–21.33
Disability (no) ***	4.45	2.31–8.54
Number of medications	1.03	0.93–1.15
Self-rated health (good) **	2.23	1.37–3.64
Level of pain *	1.19	1.02–1.38
Self-perceived age (not old)	1.01	0.89–1.16
Self-perceived memory (good) *	1.67	1.07–2.62
Self-perceived quality of life (good) ***	2.44	1.58–3.76
Self-perceived social support (yes)	1.80	0.99–3.24

* *p*-value < 0.05, ** *p*-value < 0.01, *** *p*-value < 0.001.

**Table 3:** Adjusted odds ratio of depression diagnosis.

Variable (Reference)	OR	95% Confidence Interval
Gender (men) ***	3.30	1.86–5.87
Age (70–74 years)		
75–79	1.51	0.69–3.28
80–84	1.00	0.24–4.18
85+	0.60	0.06–6.04
Region (center)		
North	1.22	0.69–2.17
South	0.68	0.39–1.17
Marital status (married)		
Single	0.81	0.32–2.08
Divorced	0.88	0.30–2.59
Widowed **	2.00	1.24–3.26
Number of chronic diseases (0)		
1–2	0.56	0.17–1.84
3+	0.64	0.19–2.20
Disability (no) *	2.11	1.10–4.10
Number of medications ***	1.23	1.10–1.38
Self-rated health (good)	1.31	0.79–2.17
Level of pain **	1.27	1.08–1.50
Self-perceived age (not old)	0.97	0.85–1.11
Self-perceived quality of life (good)	1.55	0.98–2.45

* *p*-value < 0.05, ** *p*-value < 0.01, *** *p*-value < 0.001.

## Data Availability

The data that support the findings of this study are available from the corresponding author upon reasonable request.
